# Renal angiomyolipoma with renal vein thrombosis: an incidental finding

**DOI:** 10.1259/bjrcr.20150218

**Published:** 2016-02-19

**Authors:** Elisa Melo Abreu, Teresa Margarida Cunha

**Affiliations:** Department of Radiology, Instituto Português de Oncologia de Lisboa Francisco Gentil, Lisbon, Portugal

## Abstract

A 66-year-old female with history of endometrioid endometrial carcinoma was admitted to our institution with abdominal and pelvic pain. A CT scan revealed a mass within the right upper kidney with a tumour thrombus that extended through the right renal vein up to the point of confluence with the inferior vena cava (IVC). The imaging features of the mass strongly suggested a diagnosis of renal angiomyolipoma (AML) with renal vein thrombosis. The patient was proposed an open radical right nephrectomy with right renal thrombectomy for histopathological confirmation of the diagnosis of AML with extension to the right renal vein and preventing complications such as potentially fatal pulmonary thromboembolism. The implantation of a temporary IVC filter before surgery was recommended.

## Clinical presentation

A 66-year-old female with a history of endometrioid endometrial carcinoma was sent to our institution with abdominal and pelvic pain. The physical examination and laboratory results were unremarkable. The creatinine level was 0.81 mg dl^–1^. A contrast-enhanced abdominal and pelvic CT scan was performed that revealed a mass with a fatty density within the right upper kidney and a tumour thrombus that extended through the right renal vein up to the point of confluence with the inferior vena cava (IVC).

## Differential diagnoses

- renal angiomyolipoma (AML)
- perirenal liposarcoma
- renal cell carcinoma (RCC)
- renal oncocytoma.

## Investigation/imaging findings

**Figure 1.**
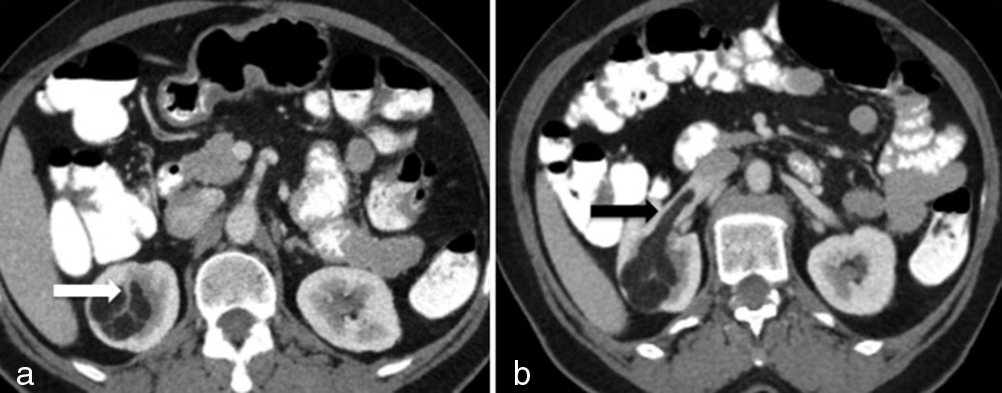
Contrast-enhanced axial CT images (a, b) show a right renal mass with fat attenuation causing a renal parenchymal defect. The mass contains visible tumour vessels extending into the renal parenchyma, a sign known as renal parenchymal vascular pedicle (white arrow; a). The fatty tumour thrombus filling the right renal vein (black arrow; b) is noteworthy.

A contrast-enhanced CT scan revealed a mass with a fatty density within the right upper kidney causing a renal parenchymal defect. The mass showed vessels extending into the renal cortex, a sign known as “renal parenchymal vascular pedicle” (Figure 1a), and a fatty tumour thrombus invading the right renal vein and extending up to the point of the confluence with IVC (Figure 1b). There were no other masses or enlarged lymph nodes.

## Treatment

The patient was proposed an open radical right nephrectomy and renal thrombectomy for histopathological confirmation of the diagnosis of AML with extension to the right renal vein and prevention of potential complications such as fatal pulmonary thromboembolism owing to renal vein invasion. Because of invasion of the renal vein, it was deemed that implanting an IVC filter before surgery would be useful in preventing an iatrogenic pulmonary thromboembolism. The use of an IVC temporary filter avoids the potential side effects of permanent filters.

## Outcome, follow-up and discussion

AML is the most common benign renal mesenchymal neoplasm arising from the perivascular epithelioid cells and is composed of a variable proportion of dystrophic vessels, smooth muscle and adipose tissue.^1^ Although it may be either sporadic or seen in association with tuberous sclerosis, the sporadic form accounts for 80–90% of cases and is most commonly found in middle-aged females.^1^

Rarely does AML reveal a potentially aggressive behaviour, including renal vein and IVC invasion, or regional lymph node involvement. Even though an invasion of the renal vein and the IVC is a known complication, AMLs with malignant character are extremely unusual.^1^ The large size of the tumour, central location (that enables the tumours to access the major veins more easily) and location on the right side (that may relate to the shorter and straighter course of the right renal vein) are contributing factors to the invasion of the renal vein.^1-3^

AMLs are often incidental findings on routine scans. Different imaging techniques can confidently diagnose them, particularly a CT scan.^2^ In spite of the potential aggressiveness of an AML at presentation, several CT scan features suggest the diagnosis. AML is a fat-containing tumour of the kidneys, with areas of fat attenuation (less than –20 HU) within the mass. It is highly vascular, with tumour vessels characteristically extending into or through the renal cortex, a signal that is also known as a “renal parenchymal vascular pedicle”.^1,4,5^

In contrast, the main differential diagnosis of perirenal liposarcoma typically reveals a renal hilar vascular pedicle, with tumour vessels extending from the fatty perirenal mass into the renal hilar vessels without traversing the parenchyma. As liposarcomas arise from the perinephric fat, they have blood supply and drainage distinct from tumours that arise from the renal parenchyma, such as an AML.^1,4^

On the other hand, tumours such as AML that arise from the kidney typically have a renal parenchymal defect with a rim of normal renal tissue interfacing with the lesion. In contrast, liposarcomas are perinephric tumours that arise from the retroperitoneal fat without a renal parenchymal defect.^1,5^

Although the presence of fat within a renal tumour strongly suggests the diagnosis of AML, foci of fat and calcifications have been described in unusual cases of RCC, such as fat-containing RCC with osseous metaplasia and oncocytomas, which were also included in the list of differential diagnoses.^6^

Malignancy should be suspected on the basis of following criteria: presence of calcifications; large, irregular tumour invading the perirenal or sinus fat; large necrotic tumour with small foci of fat and association with non-fatty lymph nodes or venous invasion.^6^ It should be noted that cases of AML with associated calcifications have been described in the literature.^1^ A variety of causes are responsible for these calcifications, including peripheral haemorrhage and osseous metaplasia.^1,7^

Although it does not imply malignancy or metastases, the particular case of AML invading the renal vein or IVC carries the risk of potentially fatal pulmonary thromboembolism. Therefore, urgent surgical treatment, including radical nephrectomy and tumour thrombectomy, is necessary even though the patients may remain asymptomatic.^8^ To decrease the risk of pulmonary embolism in patients with a tumour embolus, some surgeons recommend implanting the IVC filter before nephrectomy.^2^ Generally, a permanent IVC filter may cause several complications, including migration, thrombosis, filter fracture, IVC perforation and device infection.^9^ A temporary IVC filter minimizes the risk of these complications and is easier to insert and retrieve. Thus, it is recommended to place the filter before surgery to avoid these complications.^8^ A suprarenal IVC filter placement is usually reserved for cases in which infrarenal implantation is not possible owing to an inadequate length of available IVC or the presence of a clot prevents proper placement.^9^

A radical nephrectomy and tumour thrombectomy was performed, and histopathology revealed an admixture of mature adipose tissue, sheets of smooth muscle and thick-walled blood vessels of various proportions, proving the diagnosis of renal AML. No atypical or epithelioid features were found.

Further follow-up postoperatively for these tumours depend on the histopathological result. Malignant transformation has been reported and is associated with large tumours, perivascular epithelioid cells and nuclear pleomorphism or atypia.^2^

## Learning points

1. There is considerable overlap between the imaging appearance of large exophytic AML and perirenal liposarcoma; however, close attention to discriminating imaging features will enable a confident diagnosis.
2. AML invading the renal vein or IVC carries the risk of potentially fatal pulmonary thromboembolism. Urgent surgical treatment, including radical nephrectomy and tumour thrombectomy, is necessary even in asymptomatic patients.

## Consent

Written informed consent was obtained from the patient for publication of this case report, including accompanying images.
